# Prevalence of schistosome antibodies with hepatosplenic signs and symptoms among patients from Kaoma, Western Province, Zambia

**DOI:** 10.1186/1756-0500-6-344

**Published:** 2013-08-30

**Authors:** Lara Payne, Eleanor Turner-Moss, Mable Mutengo, Akwi W Asombang, Paul Kelly

**Affiliations:** 1 Faculty of Medicine, Imperial College London, South Kensington Campus, SW7 2AZ London, UK; 2 Barts and The London School of Medicine and Dentistry, Turner Street, E1 2AD London, UK; 3 University of Zambia, Lusaka University Teaching Hospital, P.O Box 50001, Lusaka, Zambia; 4 Washington University School of Medicine in St. Louis, 660 South Euclid, St Louis 63110, MO, USA

**Keywords:** Schistosomiasis, *Schistosoma mansoni*, Zambia, Seroepidemiology, ELISA, Hepatosplenic

## Abstract

**Background:**

Schistosomiasis is a major cause of morbidity and mortality, with over 200 million people infected worldwide. Eighty-five percent of cases are in Africa. The hepatosplenic form develops over time by an immune reaction to trapped *Schistosoma mansoni* eggs in the portal system leading to liver fibrosis, portal hypertension and oesophageal varices. Most patients presenting to the University Teaching Hospital in Lusaka with oesophageal varices, come from Western province, but no formal studies have been carried out in this area assessing the burden of hepatosplenic pathology. We aimed to define the extent of the problem in Kaoma district, western Zambia, and to correlate signs and symptoms with serology.

**Findings:**

A symptom questionnaire, demographic survey and physical examination was conducted amongst patients presenting to Kaoma district outpatient clinics. To assess the prevalence of *Schistosoma mansoni* infections, blood was collected and screened for the presence of *Schistosoma* antibodies using Enzyme linked immunosorbent assay (ELISA). Of the 110 patients screened, 97 (88%) were ELISA positive. Forty-six percent (51/110) reported haematochezia and 7% experienced haematemesis (8/110). On physical examination 27% (30/110) hepatomegaly and 17% (30/110) splenomegaly was observed amongst participants but there were few correlations between serology and signs/symptoms. On questioning 68% (75/110) of participants knew nothing about schistosomiasis transmission.

**Conclusions:**

Our serological and clinical data indicate a very heavy burden of schistosomiasis-related portal hypertension. Our evidence highlights a need for mass treatment in Kaoma to address and prevent extensive pathology of hepatosplenic schistosomiasis. Safe water and health education throughout Western Province are clearly also important.

## Findings

### Background

Schistosomiasis is a chronic parasitic disease causing morbidity and mortality in over 200 million people worldwide. Eighty-five percent of cases are in Africa [1]. Fifty-four million infections and over 130,000 deaths are attributable to *Schistosoma mansoni* annually [2,3]. Zambia has a population of approximately 13 million, 2 million of which have schistosomiasis [4]. The prevalence of *S. mansoni* is highest in some rural communities where access to safe water and basic sanitation is limited [5]. Most infections are asymptomatic or cause mild symptoms such as diarrhoea and abdominal pains. However, 5-15% of those infected with *S. mansoni* develop severe hepatosplenic disease [6] characterized by hepatic fibrosis, hepatosplenomegaly and portal hypertension [7,8].

The endoscopy unit in the University Teaching Hospital, Lusaka serves the whole country. In an audit over five years, it was observed that oesophageal varices are the most common cause of gastrointestinal bleeding in Zambian adults [9]. A high proportion of these patients were noted to come from Kaoma district, Western Province (Latitude:-14.8000; Longitude:24.8000). A subsequent survey [10] found 8% of all adults in Kaoma had a lifetime history of haematemesis. It was also noted that 10% had *S. mansoni* ova in stool samples, by the Kato-Katz technique, which has high specificity but low sensitivity [11]. Although surveys of schistosomiasis have indicated the disease is endemic, surveillance and mass drug administration (MDA) is focused at school-aged children and prevalence in adults is often unknown [12]. At the time of our study there was no documentation of MDA in the area and no safe water issues were being addressed. The Schistosomiasis Control Initiative plans prevalence studies and mass drug administration in this area, but their initiatives will not cover large scale ELISA testing. We used ELISA testing for the presence of *Schistosoma* antibodies in serum, which is the most sensitive method for assessing prevalence of infection [13,14]. We thought it important to highlight the extremely high seroprevalence and hepatosplenic disease observed in the adult population in the hope that public health initiatives could be expanded accordingly.

## Methods

We carried out a cross-sectional study among adults attending three out-patient departments of local health centres in the region: Nyango clinic, Luampa and Mangango hospitals. The first 110 patients presenting at these facilities over three days in August 2011 were recruited. The study population was restricted to those aged 18 years or over who had resided in Kaoma district for at least six months. All consecutive patients who met the criteria and who agreed to participate were admitted into the study irrespective of their initial presenting complaint to the general outpatient clinic.

Ethics approval was obtained from the University of Zambia Biomedical Research Ethics committee (Reference number 010-07-11). Formal permission was granted by the Ministry of Health and the Kaoma District Medical Office. Information regarding the study was explained to prospective study participants in their local language and voluntary informed consent obtained. No payment was made to test subjects but Praziquantel (a single dose of 40 mg/kg body weight) the standard treatment for schistosomiasis in Zambia was arranged for individuals who are found to be sero-positive for *Schistosoma* antibodies.

Sample size was calculated as previous work suggests that 10% of adult may have active schistosomiasis-related portal hypertension (8% of those interviewed reporting haematemesis [10]) and 86 samples will discriminate between a prevalence of 10% and 20%, with a power of 80% at a confidence level of 95%. In fact 112 participants were included and only two participants were subsequently excluded as they had not lived in the study area for the 6 months required. No samples were lost.

The presence of antibodies to *S. mansoni* was detected in participant’s serum using ELISA (Schisto 96, SciMedx, Denville, NJ). Positive samples were recorded according to the anonymous patient identifier code. Positive and negative standard control samples were run with each kit to ensure accuracy. Results were analysed using a spectrophotometer.

A questionnaire was administered by a local nurse/clinical officer in the language of the respondent. The participant was asked about symptoms of hepatosplenic disease (blood in stool and haematemesis), stream contact, history of previous infections and the treatment received. A physical examination was performed to evaluate for external signs of schistosomiasis. Community awareness and understanding of schistosomiasis was also assessed to see if further health education might be required.

Results were analysed with STATA™(Version 11.1) using a two-sided Fisher’s exact test for correlation between those who had a positive ELISA test against symptoms and risk factors.

## Results

The patients we recruited ranged from 18 to 89 years, with 43 men and 67 women. There were 36 patients from Mangango clinic, 27 from Nyango clinic (encompassing the Luena streams) and 47 from Luampa Hospital (catchment for the Luampa streams). There was no previous recorded mass drug administration in this area.

Of the 110 people tested, 97 (88%) were positive by ELISA for *Schistosoma* antibodies. Only 49% (54/110) reported previous schistosomiasis and 46% (51/110) of respondents said they had been treated.

On questioning with regards to symptoms, 7% reported haematemesis and 46% experienced blood in stool. All participants with previous haematemesis were positive for *Schistosoma* antibodies on ELISA. Twenty-seven percent showed hepatomegaly, 17% splenomegaly, and 72% pallor on examination. None of these signs or symptoms except pallor showed significant correlation with ELISA result but the majority of patients with these hepatosplenic symptoms of pathology were positive by ELISA (Table 1).

**Table 1:** Number of signs/symptoms of hepatosplenic pathology in relation to ELISA results

**Signs or symptoms of hepatosplenic pathology**	***Schistosoma*Elisa negative n (%)**	***Schistosoma*Elisa positive n (%)**	**P value (2 sided Fisher’s Exact)**	**Total number of participants with symptoms**
Pallor	13 (17)	65 (83)	0.0100	78
Hepatomegaly	6 (21)	22 (79)	0.0901	28
Splenomegaly	4 (21)	15 (79)	0.2338	19
Blood in stool	4 (8)	46 (92)	0.3755	50
Haematemesis	0	8 (100)	0.5924	8

Socio-demographics were not predictive of schistosomiasis infection. Neither sex (p = 0.56) or age of participants (p = 0.17) was significantly associated with ELISA result. Sixty-four percent of respondents had daily stream contact with either Luampa or Luena streams, although this showed no correlation with ELISA result (0.97, p = 1.00). The prevalence of *Schistosoma* antibodies in the blood of the population served by the Luampa streams was lower at 79% while the prevalence of those in the Luena catchment averaged 95%. Community awareness and understanding of schistosomiasis was poor; 75 (68%) of participants could not tell us anything about the transmission of schistosomiasis.

## Discussion

Although studies and national prevalence surveys of schistosomiasis have indicated that the disease is endemic in most parts of Zambia, the actual prevalence of the disease in adults is not known. The prevalence of hepatosplenic schistosomiasis disease is also unknown although estimates from a small survey based on questionnaires showed that at least 8% of people with *S. mansoni* have severe hepatosplenic disease [10]. We were concerned by reports of a high burden of oesophageal varices reported from Kaoma in Zambia. It was suspected that this could be attributable to *S. mansoni* but there had been no previous survey of this region.

We conducted a cross-sectional study involving a symptom questionnaire, demographic survey and physical examination amongst patients presenting to Kaoma outpatient clinics to observe evidence of hepatosplenic disease. To assess prevalence, blood collected was measured for *Schistosoma* antibodies using ELISA.

Our study was the first to determine the seroprevalence of infection along with patient awareness and hepatosplenic disease in adults in the Kaoma region of Zambia. Other studies have described endemic areas of schistosomiasis using multiple methods for diagnosis which although is more accurate it requires a high level of expertise and takes a long time [15]. We hoped for a fast turnaround of results so these could be implemented on in good time given the scale of the problem in Kaoma.

Since doing this study diagnostics have improved for *S. mansoni* with rapid diagnotic tests becoming available [16] and circulating-urine cathodic antigen being widely used in the field [17,18]. This study provided baseline information upon which PhD research work was planned and is underway to include a larger sample size and testing participants with parasitological methods along with ELISA and circulating-cathodic antigen for diagnosis.

We report very high prevalence of *Schistosoma mansoni* in a part of Western Province, Zambia, where fishing is the dominant way of life. From 110 participants, 88% were ELISA positive. However, only 49% recalled prior infection, indicating under-diagnosis. In the 46% of respondents who said they had been treated, there was wide variation in the treatment offered. Worryingly, 7% experienced haematemesis, a symptom of severe hepatosplenic pathology. With regards to hepatosplenic signs 27% of participants showed hepatomegaly, 17% had splenomegaly and 46% were symptomatic, with blood in stool.

As well as in symptomatic individuals, there was a high seroprevalance rate in those who were without hepatosplenic symptoms but this is to be expected. Typically the early stages of infection are asymptomatic. The high prevalence rate and the expectation that not all participants will have developed the signs or symptoms of late hepatosplenic disease means it is difficult to draw significant conclusions from the data obtained as although the majority of those who had symptoms were positive by ELISA that was also true of patients with no symptoms. The only sign that was significant for those positive by ELISA was pallor (Table 1), although non-specific this is worth noting for clinicians in endemic areas as combined with an appropriate history this could be the only sign of early schistosomiasis.

Of course, the signs and symptoms illicited are potentially attributable to multiple causes. But given the extremely high seroprevalence of *Schistosoma* antibodies, we assume that the majority of these late signs and symptoms of hepatosplenic pathology when found in combination are attributable to *S. mansoni* infection. Unfortunately the ELISA positivity could represent past exposure or ongoing infection with any *Schistosoma* species. Serological tests available at the time did not distinguish between different species of schistosome so it remains possible that some positive serological results may have been due to *S. haematobium*. Although this infection is common in some parts of Zambia, local laboratory data and a recent survey using urine filtration failed to detect any urinary schistosomiasis in the Kaoma area. Recently, a study by Lodh et al. was able to isolate and amplify *S. mansoni* specific DNA in urine samples collected from individuals resident in this area further confirming previous work that the area is predominately *S. mansoni* endemic [19]. A previous survey showed many positive stool samples [10], indicating a high proportion of these patients were likely to have *S. mansoni* exposure further supporting that our data reflects a true *mansoni* prevalence.

Another limitation of this study includes our sample being restricted to patients recruited in one of three medical centres of Kaoma. There may have been a selection bias of either those patients who felt they needed medical attention or a bias against those too unwell to travel from their homes. Therefore we cannot be sure that our data are representative of the general population.

Our study provides evidence for mass drug administration with praziquantel in the area as per Ministry of Health policy in hyper-endemic areas (that have over 60% prevalence of schistosomiasis). Major interventions to ensure safe water sources must be prioritized as the majority (64%) of Kaoma inhabitants have daily contact with contaminated streams. Health education is also crucial as the majority (68%) of participants could not tell us anything about the transmission of schistosomiasis. The high burden of hepatosplenic pathology certainly demonstrates the importance of improved health services in Kaoma district and prompt referral to tertiary care. Serious consideration should be given to provision of a Provincial endoscopy service, with variceal ligation and beta-blockade as therapeutic responses. A well-supported ultrasonography service would also help find patients at risk of developing oesophageal varices.

## Conclusions

Our efforts highlight an extremely high seroprevalence of schistosomiasis in this district with a high burden of undiagnosed hepatosplenic disease. Our evidence highlights the need for continuing surveillance and regular mass drug administration in line with Zambian Ministry of Health policy in hyper-endemic regions. Major interventions are required to reduce the burden of disease in this hyper-endemic community.

## Availability of supporting data

The data sets supporting the results of this article are available in the LabArchives repository.

**Raw data:**http://dx.doi.org/10.6070/H4TX3C9X.

**Data analysis: **http://dx.doi.org/10.6070/H4Q81B07.

## Competing interests

The authors declare that they have no competing interests.

## Authors’ contributions

LP, ETM and MM were involved in the planning, implementing and writing up the project. PK and AA participated in the planning of the project and writing up the paper. All authors read and approved the final manuscript.
